# Rift valley fever outbreak in Sembabule District, Uganda, December 2020

**DOI:** 10.1186/s42522-023-00092-3

**Published:** 2023-11-27

**Authors:** Freda Loy Aceng, Joshua Kayiwa, Peter Elyanu, Joseph Ojwang, Luke Nyakarahuka, Stephen Balinandi, Jayne Byakika-Tusiime, Alfred Wejuli, Julie Rebecca Harris, John Opolot

**Affiliations:** 1 Department of Integrated Epidemiology, Surveillance and Public Health Emergencies, Ministry of Health, Kampala, Uganda; 2 Uganda Public Health Emergency Operations Centre, Ministry of Health, Kampala, Uganda; 3 Baylor College of Medicine – Children’s Foundation, Kampala, Uganda; 4 Division of Global Health Protection, Centers for Disease Control and Prevention, Kampala, Uganda; 5 Uganda Virus Research Institute, Entebbe, Uganda; 6 World Health Organization, Kampala, Uganda

**Keywords:** Rift Valley Fever, Outbreak, Sembabule, Uganda, Zoonoses, One health

## Abstract

**Background:**

Rift Valley Fever (RVF) is a viral zoonosis that can cause severe haemorrhagic fevers in humans and high mortality rates and abortions in livestock. On 10 December 2020, the Uganda Ministry of Health was notified of the death of a 25-year-old male who tested RVF-positive by reverse-transcription polymerase chain reaction (RT-PCR) at the Uganda Virus Research Institute. We investigated to determine the scope of the outbreak, identify exposure factors, and institute control measures.

**Methods:**

A suspected case was acute-onset fever (or axillary temperature > 37.5 °C) and ≥ 2 of: headache, muscle or joint pain, unexpected bleeding, and any gastroenteritis symptom in a resident of Sembabule District from 1 November to 31 December 2020. A confirmed case was the detection of RVF virus nucleic acid by RT-PCR or serum IgM antibodies detected by enzyme-linked immunosorbent assay (ELISA). A suspected animal case was livestock (cattle, sheep, goats) with any history of abortion. A confirmed animal case was the detection of anti-RVF IgM antibodies by ELISA. We took blood samples from herdsmen who worked with the index case for RVF testing and conducted interviews to understand more about exposures and clinical characteristics. We reviewed medical records and conducted an active community search to identify additional suspects. Blood samples from animals on the index case’s farm and two neighbouring farms were taken for RVF testing.

**Results:**

The index case regularly drank raw cow milk. None of the seven herdsmen who worked with him nor his brother’s wife had symptoms; however, a blood sample from one herdsman was positive for anti-RVF-specific IgM and IgG. Neither the index case nor the additional confirmed case-patient slaughtered or butchered any sick/dead animals nor handled abortus; however, some of the other herdsmen did report high-risk exposures to animal body fluids and drinking raw milk. Among 55 animal samples collected (2 males and 53 females), 29 (53%) were positive for anti-RVF-IgG.

**Conclusions:**

Two human RVF cases occurred in Sembabule District during December 2020, likely caused by close interaction between infected cattle and humans. A district-wide animal serosurvey, animal vaccination, and community education on infection prevention practices campaign could inform RVF exposures and reduce disease burden.

**Supplementary Information:**

The online version contains supplementary material available at 10.1186/s42522-023-00092-3.

## Background

Rift Valley Fever (RVF) is a viral zoonosis first identified in 1931 during an epidemic among sheep on a farm in the Rift Valley of Kenya [1, 2]. In animals, RVF is transmitted by mosquitoes and blood-feeding flies [1]. RVF infection can cause major economic losses due to high mortality rates in young animals and abortions in pregnant females [1, 2]. Abortion storms in animals often precede human outbreaks of RVF [2].

The epidemiology of RVF virus is complex [3]. Although some human infections have resulted from the bite of infected mosquitoes, most develop from contact with the blood or organs of infected animals; they may also occur following ingestion of unpasteurized or uncooked milk from infected animals [1, 4, 5]. Human-to-human transmission has not been documented [6]. Veterinarians, abattoir workers, farmers, and herders are at greater risk of infection than other workers. The incubation period in humans varies from two to six days, and symptoms ranging from a mild flu-like illness to severe haemorrhagic fever last from 4 to 7 days [1]. Due to its varied and non-specific symptoms, clinical diagnosis is challenging, especially within the early stages of the disease, and only laboratory testing can provide a definitive diagnosis [1, 7]. For mild human cases, which are often of a short duration, no specific treatment is required. However, severe cases require general supportive therapy. Outbreaks have been reported in sub-Saharan Africa, North Africa, Saudi Arabia, and Yemen [1, 6].

On 10 December 2020, the Uganda Virus Research Institute (UVRI) notified the Uganda Public Health Emergency Operations Centre of the death of a 25-year-old male who had tested positive by reverse-transcription polymerase chain reaction (RT-PCR) for RVF using the protocol previously described [8]. He had been admitted to Nakasero Hospital in Kampala District on 8 December 2020, unconscious and with difficulty breathing, red eyes, haematuria, and unexplained bleeding from his nose and an injection site; he died the next day. Epidemiological investigations were carried out from 19 to 22 December 2020 in the patient’s home district of Sembabule by a team from the Ministry of Health and the Sembabule District Surveillance Focal Person, Veterinary Officer, and Laboratory Technician. We present the findings of this investigation, including the scope of the outbreak, exposure factors, and control measures instituted.

## Methods

### Location of the outbreak

**Fig. 1:**
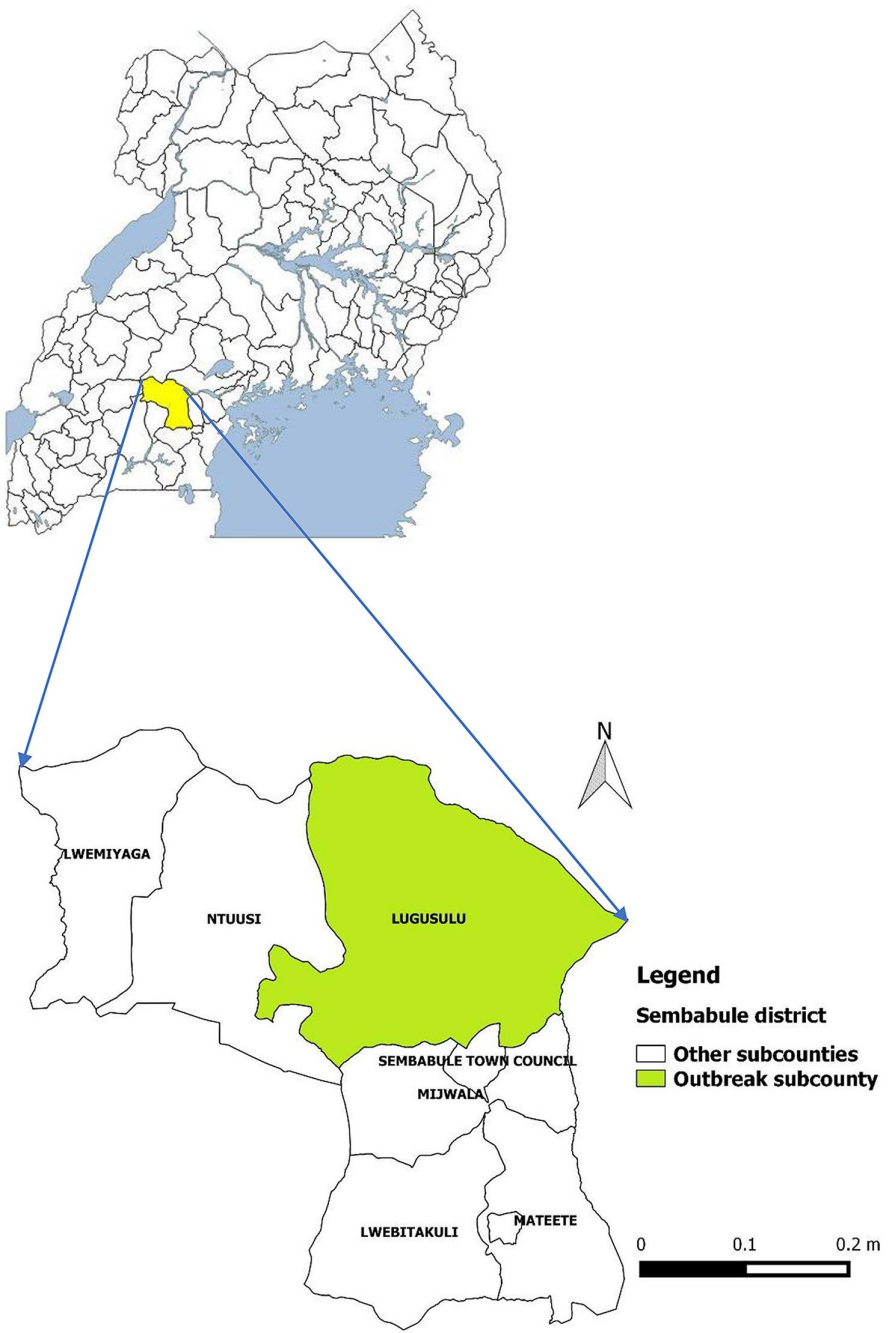
Map of Uganda showing Sembabule District

Sembabule District is a ranching and dairy farming district located in the cattle corridor of central Uganda [9, 10]. In 2020, it had a total population of 296,100 persons. The district is made of two counties and two town councils [11] (Fig. 1). The outbreak occurred in Mitima Parish, Lugusulu Sub-county, Sembabule District.

### Human case definition and case finding

A suspected case was defined as acute onset of fever (or axillary temperature > 37.5 °C) and at least two of the following symptoms: headache, muscle or joint pain, bleeding presentation, and any gastroenteritis symptoms (nausea, vomiting, abdominal pain, diarrhoea) in a resident of Sembabule District from 1 November to 31 December 2020. A confirmed case was defined as laboratory confirmation by either detection of RVF virus nucleic acid by reverse transcription–polymerase chain reaction (RT-PCR) or demonstration of serum Immunoglobulin M (IgM) antibodies by enzyme-linked immunosorbent assay (ELISA).

To identify any additional cases, we interviewed the seven herdsmen who worked with the deceased case-patient (including his brother) as well as his brother’s wife using a case investigation form. Data collected included demographic characteristics and clinical signs and symptoms from 1 November to 31 December 2020, as well as potential exposures. Exposures were identified during the week before onset (for cases) and the week before the interview (for asymptomatic cases and for other respondents). Blood samples were taken from all persons interviewed and sent to UVRI for testing. We also reviewed medical records in nearby health facilities to identify additional suspected cases. We conducted key informant interviews with district health officials to understand the outbreak situation in the district.

### Animal case definition and case finding

A suspected case was defined as any domestic animal (cattle, sheep, goat) with any past history of abortion. A confirmed animal case was defined as laboratory confirmation by detection of antibodies against RVF virus using ELISA (IgM-capture ELISA), which demonstrated recent infection as early as 6–7 days post infection.

An officer from the District Veterinary Office collected 55 blood samples from cattle, including 30 from two herds (herds A and B) that the case-patient had taken care of and 25 additional samples from two herds (herds C and D) located nearby. We included all 14 animals with a reported history of abortion. We randomly selected additional animals for testing from the remainder. We filled in an animal investigation form for each animal sampled.

### Laboratory investigations

The eight human blood samples and 55 animal blood samples were sent to the National Viral Haemorrhagic Fever Reference Laboratory at the Uganda Virus Research Institute (UVRI) for testing. Laboratory confirmation was done through ELISA detection of IgM antibodies against the RVF virus using procedures as previously described [12, 13]. In this study, IgG ELISA was performed on both human and animal samples, while IgM ELISA was performed only on human specimens. Briefly, field-collected whole blood specimens were centrifuged on arrival in the laboratory to obtain serum samples. Thereafter, samples were heat- and detergent- inactivated as previously described [14] before they were tested for anti-RVFV-specific IgM and IgG by ELISA using inactivated RVFV-infected Vero-E6 cell antigens that were supplied by the U.S. Centers for Disease Control and Prevention. Each specimen was tested at four dilution series: 1:100, 1:400, 1:1,600, and 1:6,400. The secondary antibodies used were a mouse anti-human IgG (Accurate Chemical and Scientific Corporation, NY, USA) for the human IgG ELISA, a goat anti-mouse IgG (Thermofisher Scientific, Waltham, MA USA) for the human IgM ELISA and a rabbit anti-sheep IgG (KPL, Gaithersburg, MD, USA) for animal IgG ELISA, all conjugated to horseradish peroxidase. Finally, titers and the cumulative sum optical densities of each dilution (SUMOD) minus the background absorbance of uninfected control antigen (adjusted SUMOD) were determined. Samples were deemed positive if the adjusted SUMOD was above the pre-established cut-off values of ≥ 0.45 for IgM ELISA and ≥ 0.95 for IgG ELISA.

### Analysis

Data were entered into Microsoft Excel, cleaned, and analysed using Epi info 7.2.5.0. Theme-selection analysis was done for open-ended interviews.

## Results

### Interview with the brother of Case A

**Fig. 2:**
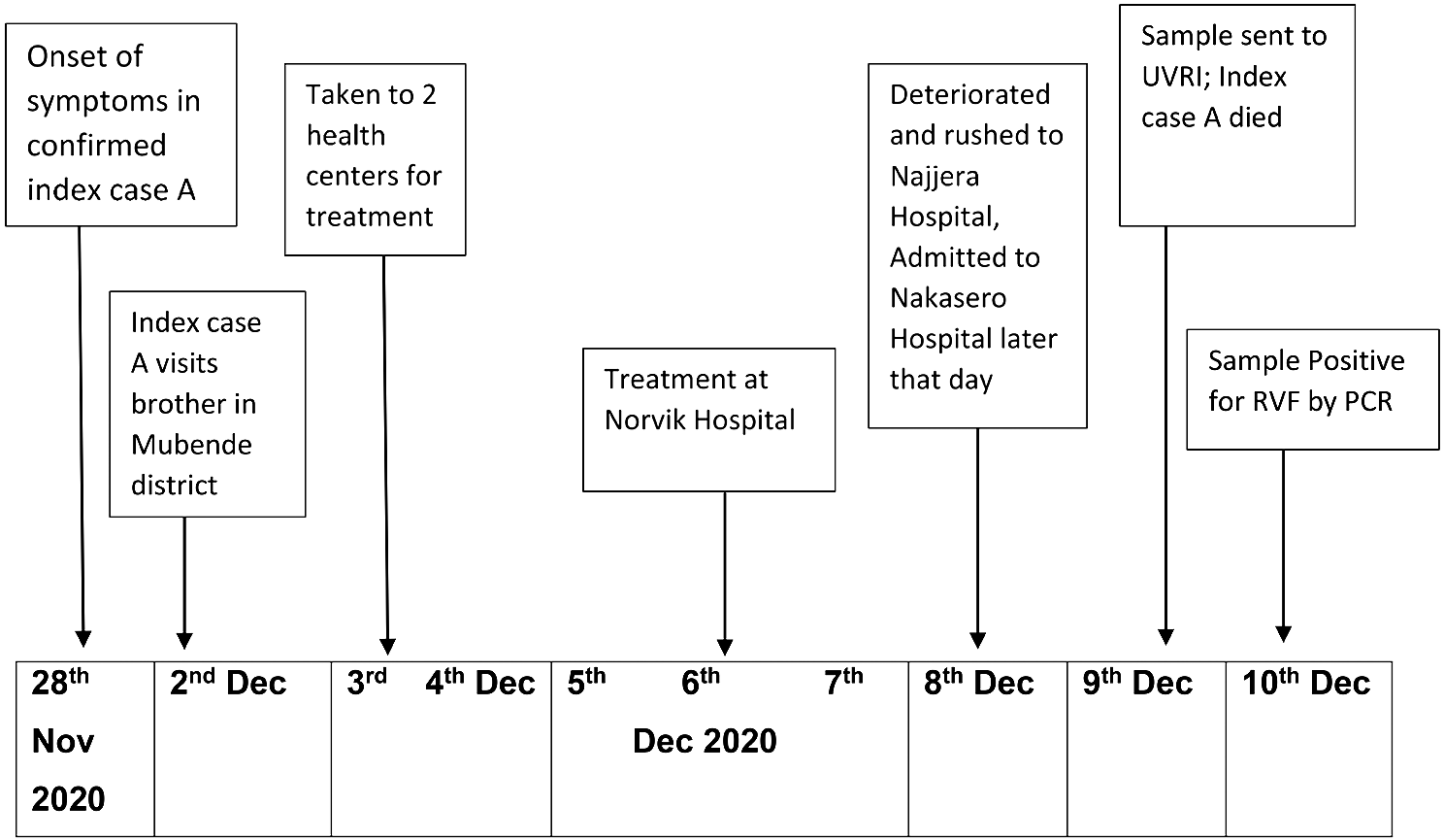
Timeline of key events for Case A during the Rift Valley Outbreak in Sembabule District

The index case (Case A), a 25-year-old cattle herder, had illness onset on November 28, 2020 while at home in Sembabule District. On December 2, Case A traveled to Mubende District to see his brother; however, he was worsening and on December 3, Case A presented to a health center in Mubende District with fever, chills, headache, and stomach pain. He was diagnosed with malaria and ulcers and given treatment (type of treatment not documented). However, his condition did not improve, and on December 4, he was taken to another health center in Mubende District where he received a similar diagnosis and was given antimalarial drugs and antacids. On December 5, Case A began vomiting blood and was taken to a private hospital in Kampala. He was diagnosed with malaria, ulcers, and an unspecified bacterial infection, based on a complete blood count. He was again provided treatment (type not known) for the ulcers, and antimalarials for the malaria. At this point, Case A was seriously ill and stopped eating. On 8 December, he began exhibiting difficulty breathing, bleeding from the nose and mouth, and persistent hiccups. He was taken to a different private hospital in Kampala where he was diagnosed with low platelets and was given unspecific intravenous infusions. The evening of the same day, his eyes turned red and he was transferred to a large referral hospital in critical condition, where he entered the Intensive Care Unit (ICU). On 9 December, Case A had a sample taken for RVF; however, he died the same day. His positive RT-PCR RVF test results were returned the next day (Fig. 2).

### Interview with the father of Case A

The father of Case A reported that since April 2020, Case A had been the full-time caretaker for a herd of approximately 1,000 cattle, which the father reported were healthy. His major activities included grazing the cattle, milking, and spraying for control of ticks and other pests. He did not participate in slaughtering nor had he handled abortus in the week before his onset. At the end of November 2020, he began feeling unwell, complaining of malaria-like symptoms including fever and headache. He was taken to a local health center in Mubende District and improved. The father reported that Case A had been diagnosed with laboratory-confirmed bovine tuberculosis three years earlier, after which he had received treatment and recovered.

### Descriptive epidemiology

#### Person and place characteristics

We interviewed the sister-in-law of the index case and the seven herdsmen with whom he worked including his brother. All the herdsmen tended the same herd of cattle as Case A; four (50%) herded goats. All animals tended to by the herdsmen drank water from a dam. Among eight human samples taken from eight respondents (excluding the index case), one tested positive for anti-RVF-specific IgG and IgM. The second case, Case B, was also a 25-year-old male herdsman; he reported no symptoms. He also reported that he had not slaughtered or butchered any animal or handled any abortus in the week before Case A’s onset. Apart from the deceased, no other respondents had signs or symptoms of RVF between 1 November and 31 December 2020.

Of the two respondents (neither were cases) who reported slaughtering, butchering animals, or handling abortus in the week before Case A’s symptom onset, neither used protective gear. Both Case A and Case B ate meat; Case A in the week before onset and Case B during the week before the interview. The deceased case and six other respondents drank unboiled milk during the same period. Neither case had direct contact (hunting, touching, or eating) with wild animals during the week in question. According to his brother, Case A had been sleeping under a mosquito net during the week before symptom onset. Two of the eight other respondents also reported that they regularly slept under a mosquito net. All seven herdsmen were involved in at least some activities known to be risk factors for RVF transmission to humans, including contact with blood and body fluids through slaughtering and butchering animals and handling abortus without protective wear, drinking raw milk, and/or not sleeping under mosquito nets.

### Animal characteristics and findings

The mean age of all 55 cattle sampled was 3.8 years (range, 1–8 years). Fifty-three (96%) were females and all were crossbreeds on a free-range production system. Of the 55 cattle, 51 (93%) were reported to be healthy; four had reportedly experienced recent signs of illness, including weight loss. Of the 53 total female cattle, 14 (26%) had any history of abortion. Animals were reportedly sprayed regularly for ticks, and no ticks were observed on the animals. Among the 55 animal samples, 29 (53%) were positive for detectable anti-RVF IgG, an indication of previous exposure (Table 1). Of the 29 positive cattle, one (3%) was male. Of the 28 IgG-positive females, 11 (39%) had a history of abortion. Of the four unhealthy cattle, only one (25%) was positive.

**Table 1:** Cattle herd ID, IgG positive, and history of abortions

Cattle herd ID	Total tested	IgG positive	History of abortion
A	18	9	1
B	12	4	2
C	11	8	2
D	14	8	6

### Environmental investigations

The domestic animals kept in the deceased’s homestead include cattle, goats, dogs, and cats. We found charcoal burning activity, a sign of logging, close to the grazing land.

The investigation team educated the deceased’s family and neighbours on Rift Valley Fever including the cause, transmission, and risk factors as well as control and prevention measures. However, some members of the family reported that Case A was bewitched.

## Discussion

This RVF outbreak comprised two confirmed case-patients, both young cattle herdsmen residing in the cattle corridor of Uganda. One of the case-patients was asymptomatic, while the other was ill, had a delayed diagnosis, and died. Both likely contracted their infection through their interactions with infected animals. This report underscores the importance of RVF surveillance in Uganda and increasing the index of suspicion for viral haemorrhagic fevers among clinicians, especially for patients with high-risk occupations or from high-risk areas.

Cattle herders are known to be a high-risk group for RVF in Uganda [15]. Both case-patients were young males, who are usually more involved in the direct handling of livestock than older herdsmen [16]. Although neither case-patient was not involved in the highest-risk activities of slaughtering, butchering, or handling abortus of animals, their regular exposure to livestock, consumption of unboiled cow milk, or exposures to infected mosquitoes all may have provided opportunities for infection [3]. Abortions among their cattle herds may have been a missed signal indicating an increased chance of RVF infections; herdsmen may also be potentially exposed if they touch aborted fetuses of infected animals [15].

The case-patient who died had a delayed diagnosis. He initially presented with nonspecific symptoms such as shivering, fever, headache, and stomach pain. In the Ugandan setting, it is easy to mistake these for malaria, typhoid, or other common diseases [17]. However, his later symptoms became more indicative of a viral haemorrhagic fever (VHF), with vomiting blood, difficulty breathing, bleeding, hiccups, red eyes, and hematuria [16, 18]. The patient had a positive malaria test by blood smear but did not respond to anti-malarial treatment. Trainings for healthcare workers on possible differential diagnoses for patients who do not respond to malaria treatment may facilitate detection and appropriate management for patients with other conditions such as RVF.

None of the persons who slaughtered or butchered animals or handled abortus used personal protective equipment (PPE). A 2016 study in Kabale District, Uganda showed that only 29% said they used PPE when handling animals [19]. The use of PPE by animal handlers may help to decrease RVF transmission during outbreaks [20]. The use of PPE in Uganda has been shown to vary by occupation; 77% of butchers used some kind of PPE, compared to 12% of farmers [19]. The cost of using PPE can be prohibitive in rural settings. Provision of subsidized PPE may be needed for such settings to protect workers.

Of the animals sampled, slightly more than half had detectable anti-RVF IgG, an indication of previous exposure and infection. Of the positive females in this study, nearly four in ten had a history of spontaneous abortion. While we cannot conclusively link the abortions to RVF infection, it is suggestive.

We identified possible logging in the affected area. Deforestation results in an increase in habitat availability for mosquitoes which may increase mosquito breeding. The removal of trees takes away a crucial store of moisture that causes water to pool and get heated by direct sunlight, providing suitable conditions for mosquitoes to breed. Deforestation has been shown previously to increase malaria prevalence in malaria-endemic countries throughout the tropics [21], and may similarly have provided habitats for RVF-carrying mosquitoes in this area.

The high rates of RVF positivity among animals in this area point to the importance of implementing control measures among the animal population. A 2019 study in Mayotte Island suggested that reactive animal vaccination during an outbreak could be the most effective control measure for RVF, leading to the prevention of both human and livestock cases, and would require a smaller number of vaccine doses than vaccination at a later time in order to result in a reduction of human cases [5]. Disease surveillance in animals, contingency planning, and timely animal vaccination are important aspects for decreasing human disease risk [5].

The primary limitation of the study was the self-reporting of exposures and symptoms, which can be compromised by recall bias.

## Conclusions

In conclusion, an RVF outbreak in Sembabule district was likely facilitated by close interaction between infected animals and humans. A district-wide serosurvey and ongoing surveillance could provide important information on the prevalence and trends of RVF in animals. Animal vaccination may be warranted, as well as community education on prevention measures against RVF.

### Electronic supplementary material

Below is the link to the electronic supplementary material.

Supplementary Material 1

## Data Availability

The datasets used and analyzed during this study belong to the Uganda Ministry of Health and are available from the corresponding author on reasonable request and upon permission from the Ministry.

## Abbreviations

ICU: Intensive Care Unit
MOH: Ministry of Health
PCR: Polymerase Chain Reaction
PPE: Personal Protective Equipment
RVF: Rift Valley Fever
UVRI: Uganda Virus Research Institute
