# Correction: Rift valley fever outbreak in Sembabule District, Uganda, December 2020

**DOI:** 10.1186/s42522-023-00095-0

**Published:** 2024-01-02

**Authors:** Freda Loy Aceng, Joshua Kayiwa, Peter Elyanu, Joseph Ojwang, Luke Nyakarahuka, Stephen Balinandi, Jayne Byakika-Tusiime, Alfred Wejuli, Julie Rebecca Harris, John Opolot

**Affiliations:** 1 Department of Integrated Epidemiology, Surveillance and Public Health Emergencies, Ministry of Health, Kampala, Uganda; 2 Uganda Public Health Emergency Operations Centre, Ministry of Health, Kampala, Uganda; 3 Baylor College of Medicine – Children’s Foundation, Kampala, Uganda; 4 Division of Global Health Protection, Centers for Disease Control and Prevention, Kampala, Uganda; 5 Uganda Virus Research Institute, Entebbe, Uganda; 6 World Health Organization, Kampala, Uganda

**Correction: One Health Outlook 5, 16 (2023)**

**https://doi.org/10.1186/s42522-023-00092-3**

After publication of this article [1], the authors reported that in this article the graphics relating to Figs. 1 and 2 captions had been interchanged; the figures should have appeared as shown below.

**Fig. 1:**
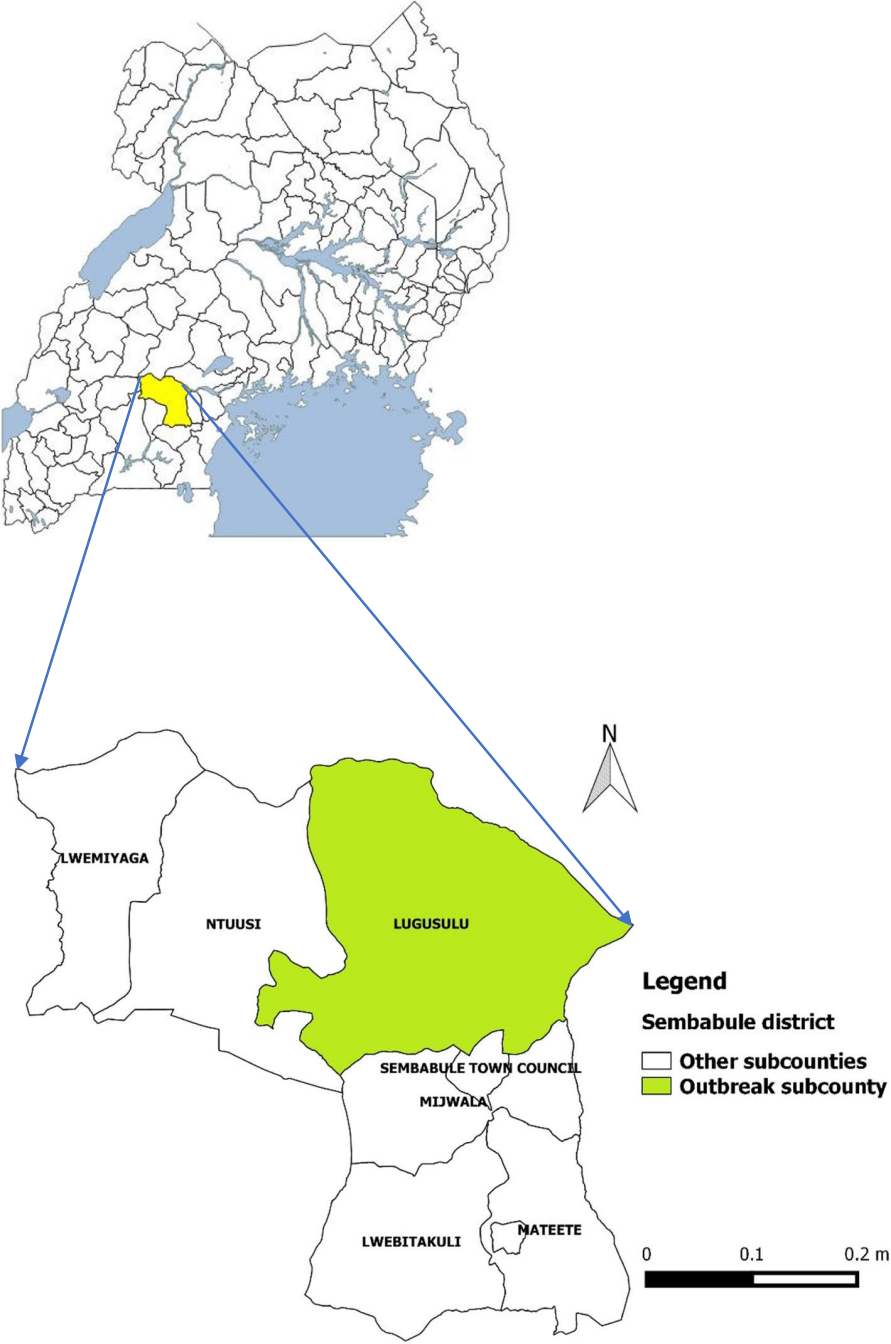
Map of Uganda showing Sembabule District

**Fig. 2:**
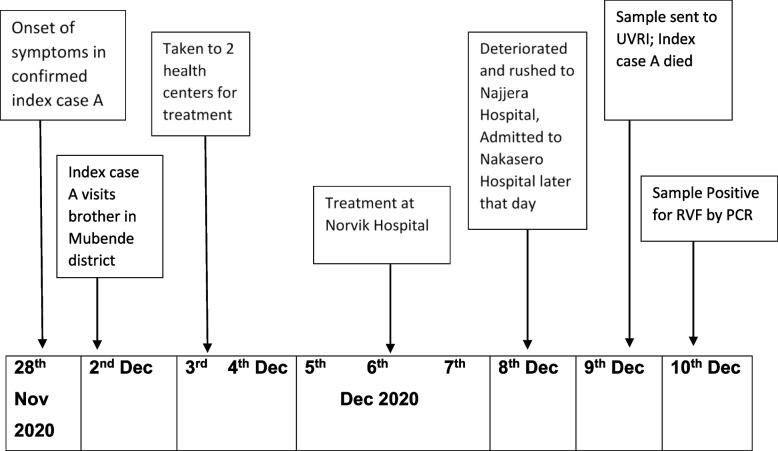
Timeline of key events for Case A during the Rift Valley Outbreak in Sembabule District

The original article [1] has been corrected.

## References

[1] Aceng FL, Kayiwa J, Elyanu P (2023). Rift valley fever outbreak in Sembabule District, Uganda, December 2020. One Health Outlook.

