# Supplementary material for: Rift valley fever outbreak in Sembabule District, Uganda, December 2020
Source: One Health Outlook. 2023 Nov 27;5:16. doi: 10.1186/s42522-023-00092-3 (PMC10680244; doi:10.1186/s42522-023-00092-3)
Supplement: Supplementary file 1 — Supplementary Material 1 [file 42522_2023_92_MOESM1_ESM.pdf]

RIFT VALLEY FEVER IN SEMBABULE DISTRICT, 2020
CASE CONTROL STUDY QUESTIONNAIRE

ID: CASE ☐ CONTROL☐

Section 1. Demographic

Patient's Surname: ________________ Other Names:____________________________ Age: _______ ☐ Years ☐ Months
Gender: ☐ Male ☐ Female Phone Number of Patient/Family Member:_____________________
Owner of Phone: ________________

Status of Patient at Time of This Case Report: ☐ Alive ☐ Dead *If dead,* Date of Death: ___/___/____ (D, M, Yr)

Permanent Residence:
Head of Household: _________________________ Village/Town: ________________________ Parish: _________________________
Sub-County: ____________________________ District: ____________________________
Country of Residence: _________________

Occupation:
☐ Farmer ☐ Butcher ☐ Hunter/trader of game meat ☐ Miner ☐ Herdsman ☐ Housewife ☐ Pupil/student
☐ Child ☐ Religious leader
☐ Businessman/woman; type of business: ___________________ ☐ Transporter; type of transport: ______________________
☐ Healthcare worker; position: ____________________ healthcare facility: _____________________ ☐ Traditional healer
☐ Other; please specify occupation: ____

Location Where Patient Became Ill:
Village/Town: _________________________ Sub-County: _________________________ District: _______________________
GPS Coordinates at House: latitude: __________________ longitude: ________________________
If different from permanent residence, Dates residing at this location: ___/___/____ - ___/___/____ (D, M, Yr)

Section 2. Clinical Signs and Symptoms

Date of Initial Symptom Onset: ____/____/______ **(D, M, Yr)**

Please tick an answer for ALL symptoms indicating if they occurred during this illness between symptom onset and case detection:

Symptom	Answer
Fever	☐ Yes ☐ No ☐ Unk
If yes, Temp: ____º C Source: ☐ Axillary ☐ Oral ☐ Rectal	
Vomiting/nausea	☐ Yes ☐ No ☐ Unk
Diarrhea	☐ Yes ☐ No ☐ Unk
Intense fatigue/general weakness	☐ Yes ☐ No ☐ Unk
Anorexia/loss of appetite	☐ Yes ☐ No ☐ Unk
Abdominal pain	☐ Yes ☐ No ☐ Unk
Chest pain	☐ Yes ☐ No ☐ Unk
Muscle pain	☐ Yes ☐ No ☐ Unk
Joint pain	☐ Yes ☐ No ☐ Unk
Headache	☐ Yes ☐ No ☐ Unk
Cough	☐ Yes ☐ No ☐ Unk
Difficulty breathing	☐ Yes ☐ No ☐ Unk
Difficulty swallowing	☐ Yes ☐ No ☐ Unk
Sore throat	☐ Yes ☐ No ☐ Unk
Jaundice (yellow eyes/gums/skin)	☐ Yes ☐ No ☐ Unk
Conjunctivitis (red eyes)	☐ Yes ☐ No ☐ Unk
Skin rash	☐ Yes ☐ No ☐ Unk
Hiccups	☐ Yes ☐ No ☐ Unk
Pain behind eyes/sensitive to light	☐ Yes ☐ No ☐ Unk
Coma/unconscious	☐ Yes ☐ No ☐ Unk
Confused or disoriented	☐ Yes ☐ No ☐ Unk

Symptom	Answer
**Unexplained bleeding from any site**	☐ Yes ☐ No ☐ Unk
If Yes:	
Bleeding of the gums	☐ Yes ☐ No ☐ Unk
Bleeding from injection site	☐ Yes ☐ No ☐ Unk
Nose bleed (epistaxis)	☐ Yes ☐ No ☐ Unk
Bloody or black stools (melena)	☐ Yes ☐ No ☐ Unk
Blood or "coffee grounds" in vomit (hematemesis)	☐ Yes ☐ No ☐ Unk
Coughing up blood (hemoptysis)	☐ Yes ☐ No ☐ Unk
Bleeding from vagina, other than menstruation	☐ Yes ☐ No ☐ Unk
Bruising of the skin (petechiae/ecchymosis)	☐ Yes ☐ No ☐ Unk
Blood in urine (hematuria)	☐ Yes ☐ No ☐ Unk
Other hemorrhagic symptoms	☐ Yes ☐ No ☐ Unk

If yes, please specify: ____________________________

Other non-hemorrhagic clinical symptoms: ☐ Yes ☐ No ☐ U
If yes, please specifiy: ____________________________

Section 3. Hospitalization information

At the time of this case report, is the patient hospitalized or currently being admitted to the hospital? ☐ Yes ☐ No

If yes, Date of Hospital Admission: ____/____/_____ (dd, mm, yyyy) Health Facility Name: ___________________________________

Village/Town: _________________________ Sub-County: ________________________ District: _______________________

Is the patient in isolation or currently being placed there? ☐ Yes ☐ No *If yes,* date of isolation: ____/____/_____ (dd, mm, yyyy)

Was the patient hospitalized or did he/she visit a health clinic previously for this illness? ☐ Yes ☐ No ☐ Unknown

If yes, please complete a line of information for each previous hospitalization:

Dates of Hospitalization	Health Facility Name	Village	District	Was the patient isolated?
___/___/____ - ___/___/____ (*dd, mm, yyyy*)				☐ Yes<br>☐ No
___/___/____ - ___/___/____ (*dd, mm, yyyy*)				☐ Yes<br>☐ No

Section 4. Epidemiological Risk Factors and Exposures

1. Do you have any animals in your household? Yes [ ] No [ ]

If yes, which ones? (list them…)

Cows Yes [ ] No [ ]
Goats Yes [ ] No [ ]
Sheep Yes [ ] No [ ]
Pigs Yes [ ] No [ ]
Dogs Yes [ ] No [ ]
Other Yes [ ] No [ ]
Specify if other ____

2. If Yes, do you herd these animals?
   Cows Yes [ ] No [ ]
   Goats Yes [ ] No [ ]
   Sheep Yes [ ] No [ ]
   Pigs Yes [ ] No [ ]
   Dogs Yes [ ] No [ ]
   Other Yes [ ] No [ ]
3. Do you usually share the same shelter with animals at night? Yes [ ] No [ ]
4. Where do you normally take your animals to drink water?
   a. Swamp Yes [ ] No [ ]
   b. River Yes [ ] No [ ]
   c. Lake Yes [ ] No [ ]
   d. Well Yes [ ] No [ ]
   e. Dam Yes [ ] No [ ]
   f. Other [Specify]………
5. Where do you normally take your animals to graze?
   a. Swamp Yes [ ] No [ ]
   b. River Yes [ ] No [ ]
   c. Lake Yes [ ] No [ ]
   d. Well Yes [ ] No [ ]
   e. Dam Yes [ ] No [ ]
   f. Other [Specify]………

IN THE PAST ONE(1) WEEK PRIOR TO SYMPTOM ONSET: (Remember IP for RVF in humans ranges from 2-6 days)

6. Did you slaughter any animal/animals in the past one week prior to symptom onset ?
7. Did you participate in slaughter of any sick animal in the past one week prior to symptom onset? (okubaaga)
   Yes [ ] No [ ]
8. Did you participate in slaughter of any dead animal of unknown cause of death in the past one week prior to symptom onset? (okubaaga)
   Yes [ ] No[ ]
9. Did you butcher any animal/animals in the past one week prior to symptom onset?
   Yes [ ] No [ ]
10. Did you participate in butchering of any meat from an animal of unknown cause of death in the past one week prior to symptom onset? (okutematema)
    Yes [ ] No [ ] Don't know [ ]
11. Did you participate in butchering of any meat from an animal of unknown origin in the past one week prior to symptom

onset? (okutematema) Yes [ ] No [ ] Don't know [ ]

12. Did you handle any abortus in the past one week prior to symptom onset? Yes [ ] No [ ]
13. Did you wear any protective gear? Yes [ ] No [ ]
    a. Slaughtering Yes [ ] No [ ]
    b. Butchering Yes [ ] No [ ]
    c. Handling abortus Yes [ ] No [ ]
    d. Other______________________________

    Specify if other___________________________
14. Did you buy meat from butchery in the past one week prior to symptom onset? Yes [ ] No [ ]
15. How much did you buy a kg of this meat on average?_________________(UGX)
16. Did you touch this uncooked meat? Yes [ ] No [ ]
17. Did you eat any meat in the past one week prior to symptom onset? Yes [ ] No [ ]
18. If yes, which form of meat did you eat?
    a. Boiled Yes [ ] No [ ]
    b. Roasted Yes [ ] No [ ]
    c. Fried Yes [ ] No [ ]
    d. Fried and Boiled Yes [ ] No [ ]
    e. Roasted and Fried Yes [ ] No [ ]
19. If boiled, for how long?_____________ [Minutes]
20. Did you milk animals in the past one week prior to symptom onset? Yes [ ] No [ ]
21. Did you take unboiled milk in the past one week prior to symptom onset? Yes [ ] No [ ]
22. Did you travel outside your home or village/town in the past one week prior to symptom onset?

    Yes [ ] No [ ] Unknown [ ]

If yes, Village: ___________________ District: ______________________ Date(s): ___/___/____ - ___/___/____ (D, M, Yr)

23. What were the reasons for travelling?
24. Did the patient have direct contact (hunt, touch, eat) wild animals in the past one week prior to symptom onset?

    Yes [ ] No [ ] Unknown [ ]

    Bats Yes [ ] No [ ] Unknown [ ]

    Primates e.g. Monkeys Yes [ ] No [ ] Unknown [ ]

    Rodents e.g guinea pigs Yes [ ] No [ ] Unknown [ ]

    Snakes Yes [ ] No [ ] Unknown [ ]

    Wild birds Yes [ ] No [ ] Unknown [ ]

    Other Yes [ ] No [ ] Unknown [ ]

    Specify if other____
25. Have you been sleeping under a mosquito net in the past one week prior to symptom onset? Yes [ ] No [ ]
26. Are there some activities that destroy the environment going on around?

    List them __________________________
27. Are there mosquitoes in the environment? Yes [ ] No [ ] Unknown [ ]
28. Is there standing water (swamps, water pools) in the environment? Yes [ ] No [ ]
29. Is there a forest nearby? Yes [ ] No [ ]

Case Investigation Form completed by:

Name: ______________________________ Phone: __________________________ E-mail: ________________________

Position: _____________________________
